# Genes, shells, and AI: using computer vision to detect cryptic morphological divergence between genetically distinct populations of limpets

**DOI:** 10.1038/s41598-025-30613-1

**Published:** 2025-12-12

**Authors:** Jack D. Hollister, David A. Paz-García, Rodrigo Beas-Luna, Tammy Horton, Xiaohao Cai, Phillip B. Fenberg

**Affiliations:** 1 School of Ocean and Earth Science, National Oceanography Centre, University of Southampton, Waterfront Campus, European Way, Southampton, SO14 3ZH UK; 2 Natural History Museum, London, Cromwell Road, South Kensington, London, SW7 5BD UK; 3 Centro de Investigaciones Biológicas del Noroeste (CIBNOR), Conservation Genetics Laboratory, Playa Palo de Santa Rita Sur, IPN Street 195, 23096 La Paz, Baja California Sur Mexico; 4 Faculty of Marine Sciences, Autonomous University of Baja California, Ensenada, Baja California Mexico; 5 National Laboratory of Climate Change Biology, SECIHTI, Mexico City, Mexico; 6 National Oceanography Centre, European Way, Southampton, SO14 3ZH UK; 7 School of Electronics and Computer Science, University of Southampton, University Road, Southampton, SO17 1BJ UK

**Keywords:** Computational biology and bioinformatics, Ecology, Ecology, Evolution, Genetics, Zoology

## Abstract

Many species are composed of two or more genetically distinct clades, indicating ongoing or past evolutionary divergence. Often however, there are no obvious morphological differences between clades, making it difficult to accurately assess specific aspects of biodiversity or to enact targeted conservation efforts. New advancements in artificial intelligence tools can be used to categorise individuals into their respective genetic clades and to highlight their distinguishing morphological characters that would otherwise be hidden from human observers. Here, we applied computer vision and explainable artificial intelligence techniques to four limpet species that display well-defined phylogeographic breaks along the Baja California and California coasts. A fine-tuned convolutional network, trained and evaluated over 100 resampling iterations, classified individuals into their genetic clades with median F1-scores of up to 0.96. F1-score performance was markedly higher for true clade groups than the controlled mixed-groups, confirming the presence of features specific to the clades. Saliency maps consistently emphasised structures such as the keyhole in *Fissurella volcano* and the ridge tips in *Lottia conus* as distinguishing features, and subsequent shape analyses confirmed significant divergence between clades. These results demonstrate the power of computer vision and explainable artificial intelligence to expose otherwise cryptic morphological diversity and provide a scalable, reproducible workflow that can broaden the biodiversity toolkit and refine eco-evolutionary research across taxa.

**Supplementary Information:**

The online version contains supplementary material available at 10.1038/s41598-025-30613-1.

## Introduction

Understanding the processes that generate and maintain biodiversity between and within species is a central aim of ecology and evolutionary biology^1^. Although genetics, morphology, or a combination of the two are routinely used to delimit taxa and populations, a substantial fraction of diversity remains hidden because genetically distinct lineages may be morphologically indistinguishable, known as cryptic divergence. In such cases, the human eye cannot easily discriminate external traits, and molecular markers are often relied on to provide a diagnostic tool for classifying groups^2^.

The prevalence of cryptic divergence across the animal kingdom has become increasingly apparent with advances in molecular techniques. A comprehensive meta-analysis of 2,207 cryptic species across major metazoan taxa and biogeographical regions^3^, revealed that cryptic species are homogeneously distributed among taxonomic groups rather than concentrated in particular lineages or environments. This finding suggests that morphological stasis upon speciation is seemingly common, independent of phylogenetic relationships or ecological circumstances, and indicates that cryptic diversity predictably affects biodiversity estimates across all animal groups.

There are also high levels of cryptic divergence within species, where there are no obvious morphological differences between genetically distinct populations^4^. If there are any morphological differences between cryptically divergent populations within species, it is likely harder to notice them compared to differences between completely separate species, because the genetic differences are not as significant^5^. Given the accelerating loss of species and populations under human impacts, accurate and reproducible methods are urgently needed for detecting morphological differences between cryptically divergent populations or species to inform conservation efforts and provide eco-evolutionary biologists with better tools for understanding genotype-phenotype interactions.

Explainable artificial intelligence (XAI) are techniques that allow for AI model outputs to be reviewed and understood by humans^6^. XAI have been particularly advanced within the subfield of computer vision (CV), which now offer a tractable solution to the challenges faced in identifying differences in morphology between cryptic groups^7^. Heatmaps, a form of CV XAI, provide a visual representation of the regions within an image that most strongly influence model prediction^8^. They generate an overlay in which the individual pixels, or spatial areas of pixels, that contribute most to classification decisions are highlighted. One such technique, known as *saliency mapping*, assigns a score to each pixel according to its importance in the final output decision^9–11^. The scored pixels are then rendered in a colour scale, where ‘hotter’ colours indicate higher importance. These maps therefore serve as a crucial link between model prediction and human interpretation, enhancing the transparency and reliability of automated morphological assessments.

A trained convolutional neural network (CNN) can detect minute shape or colour variations between closely related species, differences which can often elude human observers^12^. Furthermore, heatmap systems reveal the pixels most responsible for each classification^13^. By coupling automated classification with feature visualization tools, researchers can examine and objectively measure morphological differences, enhancing our understanding of cryptic patterns of biodiversity.

In this study, we developed a CV pipeline to examine four species of limpets with significant population genetic differences along the coasts of Baja California and California^14,15^. Despite clear genetic distinctions between clades, there are no outward morphological differences that have been noted, either in the literature or from field observations of the co-authors, suggesting cryptic divergence. Our primary goal was to train a CNN to accurately classify shells to their respective genetic clades. Next, we aimed to uncover specific features that contribute to this accuracy using saliency maps, which helped guide our analyses and interpretation of shell shape differences between clades. These results can then be used to hypothesise on the potential eco-evolutionary reasons for the morphological differences between clades. Using our explainable AI pipeline, researchers will be in a better position to explore phenotypic differences between cryptic groups and their ecological and evolutionary significance.

## Methods

### Species selection and genetic clade classification

The following four limpet species were analysed: *Fissurella volcano*^16^*Lottia conus*^17^*Lottia gigantea*^18^ and *Lottia strigatella*^19^. The *Lottia* species are members of the Patellogastropoda (true limpets) whereas *F. volcano* belongs to the Fissurellidae (keyhole limpets). Despite their superficial morphological similarities, keyhole limpets and true limpets are not closely related phylogenetically. Fissurellidae can be easily distinguished from the true limpets by the presence of their keyhole. Between *Lottia* species, *L. gigantea* can be easily distinguished from the others due to its large size difference. While there can be some confusion between *L. conus* and *L. strigatella*^13^ author PBF is an expert in *Lottia* species from the Baja California Peninsula and is able to visually differentiate all species in this study. Within species however, there is high morphological variability^13^.

Specimens were classified into their predefined genetic groups based on previous molecular studies which defined distinct genetic clades separated by clear phylogeographic breaks. In *F. volcano*, *L. conus*, and *L. strigatella*, major phylogeographic breaks along the western Baja California Peninsula was identified using the mitochondrial marker *CO1*^15^. By contrast, *L. gigantea* shows no breaks with *CO1* or microsatellites^20^ but does exhibit two breaks identified by genome-wide SNP analysis^14^.

Throughout this study, populations north of their respective breaks are termed the Northern clade and populations south of the breaks, the Southern clade. For *L. gigantea* we restricted sampling to specimens spanning the Californian break because material from the more southerly clade is scarce. These clade designations provided the framework for all subsequent morphological and CV analyses.

### Specimen collection

Specimens were obtained from two primary sources: field sampling and natural history collections. This dual approach provided a comprehensive sample across phylogeographic breaks while accommodating logistical constraints that limited field sampling at every location. Field collections yielded representatives of the Northern and Southern clades of *F. volcano, L. conus* and *L. strigatella*. Additional specimens were sourced from the Natural History Museum of Los Angeles County (LACM), comprising both clades of *F. volcano, L. conus* and *L. gigantea*. All museum material had been morphologically identified by museum taxonomic experts. Specimen counts were: *F. volcano* = 552 (181 Northern and 371 Southern); *L. conus* = 974 (345 Northern and 629 Southern); *L. gigantea* = 352 (162 Northern and 190 Southern); *L. strigatella* = 789 (215 Northern and 574 Southern). Location counts are provided in the supplementary Data (Sup. Table 1).

Each specimen was photographed in dorsal and ventral orientations using a Panasonic Lumix DC-G9 with an OM SYSTEM 90 mm macro lens on a black background under standardised lighting and magnification. Between three and twenty-one photographs (depending on specimen size) were captured per specimen and focus-stacked using Helicon Focus software to produce a single high-resolution image with consistent depth of field for subsequent CV analyses. All shells, whether obtained from field collections or museum holdings, were dry and photographed under identical imaging conditions (camera, distance, angle, background, and lighting) to ensure consistency across sources. Dry shells are generally stable under standard museum storage conditions, and no published reports describe substantive morphological alteration of dry-stored limpet shells over time, although minor surface variation between sources cannot be excluded.

### Model selection and configuration

For image classification, we employed the VGG16 neural network architecture^21^, initially trained on the ImageNet dataset^22^. Custom top layers were added to adapt the model to the specific requirements of this study. Following preliminary testing, hyperparameters were tuned to optimise performance across all species and orientations. The optimised parameters were consistently applied throughout the pipeline. To ensure reproducibility, the pipeline was seeded with a fixed random state. To ensure the robustness of our findings and to verify that the results were not influenced by random chance, each classification configuration was repeated 100 times. In each iteration, the pipeline randomly sampled training (set to 120 images per class) and validation (set to 30 images per class) images from the total pool of available images required for classifier construction from each respective class (Northern and Southern). The remaining images were reserved as test data for evaluation purposes, referred to as ‘Test-full’. To address potential class imbalance within the test dataset, a further subset was evenly sampled across all classes, referred to as ‘Even-test’ (set to 20 images per class). To address potential sub-class imbalance within the clades, where locations that had large numbers have the potential to create location-specific morphological features rather than the desired clade-specific morphological features, we set a limit of 100 maximum specimens per location. We randomly sampled 100 specimens from these locations and put the remainder into the test sets. Images assigned to training and validation batches were augmented to generate synthetic images. This augmentation process, which included operations such as rotation, flipping, and scaling, is well-documented to enhance the performance potential of image classifiers by increasing the diversity of the training dataset^23,24^.

### Mixed-group validation

To provide a control group, we trained a parallel set of mixed-group models. All network settings were identical to the original clade-based models. For each species, its two respective clade labels were replaced by two synthetic classes created through random mixing: every mixed group contained equal numbers of Northern and Southern images. Each mixed class therefore represented a uniform distribution of the original categories, so any clade-specific signal should be removed.

Performance on these control models serves as a benchmark for model behaviour for several critical reasons established in recent deep learning research. Systematic experiments demonstrated that convolutional networks can easily fit random labelling of training data, achieving near-perfect training accuracy even when no meaningful relationship exists between images and labels^25^. However, while these networks could memorize the random associations during training, they achieved test performance no better than random chance, producing an accuracy of ~10% on the 10-class CIFAR-10 dataset^25^. This demonstrates that networks learning from natural data with genuine structure behave qualitatively differently from those fitting arbitrary random associations. While deep networks are capable of memorising noisy data, they tend to prioritize learning simple patterns first, and that networks behave differently when learning from structured versus random data^26^. This preferential learning of meaningful patterns when genuine structure exists provides the theoretical foundation for using mixed-group controls to distinguish real clade specific signals from spurious correlations.

If our CNN truly exploits clade-specific morphological features, its accuracy could reach high levels on the original task but fall to chance levels (~50% for two-class problems) on the mixed-group task. If there are no real morphological differences between groups, both the original and mixed-group models will perform similarly, with moderate accuracy. This is because the classifier will pick up on false signals caused by factors like imaging differences, batch effects, or technical issues, rather than true clade differences. The mixed-group control effectively tests whether the model is identifying genuine clade-specific features rather than simply memorising arbitrary training examples^27–30^.

### Size differentiation

Because shell outline and erosion can vary with specimen size^31,32^, any systematic size difference between clades could bias the CV models. To test for such bias, we measured the major-axis length of every shell with digital calipers (mm) and compared size distributions between Northern and Southern clades within each species using the Mann–Whitney U test. Size ranges overlapped broadly in all cases and none of the pairwise comparisons were significant (P >0.05). Limpets are generally not known to exhibit external sexual dimorphism^33^ unless they have protandric hermaphrotidism^34^. *Lottia gigantea* shows size-related sexual dimorphism linked to protandrous sex change but there are no shell characteristics that distinguish the sexes^35^. However, we included a broad range of shell sizes within each clade but excluded the largest specimens and those with heavy erosion, aiming to minimise any potential influence of sex-related and erosion variation in this species. We therefore assume that size-related cues, including those arising from sexual dimorphism, are unlikely to confound the classifier.

### Model attention interrogation & shape analysis

Previous work shows that CNNs trained on morphological datasets can reveal the image regions most diagnostic for classification via XAI heatmaps^13^. The SmoothGrad saliency algorithm was used for this study^36^. Preliminary runs confirmed that the saliency maps centred on the specimens rather than the background, validating their use for downstream analysis.

Guided by these maps, we carried out a mask-based shape analysis. Object regions were first detected with YOLOv8^37^ and then precisely segmented with the Segment Anything model^38^. All masks were rescaled to the same major-axis length (1.0) while preserving aspect ratio, ensuring that subsequent metrics captured shape rather than size. The metrics extracted were:

1. **Circularity -** equals 1 for a perfect circle and declines as outlines become more irregular, defined as: $Circularity= \frac{4\pi x Area}{({Perimeter}^{2})}$
2. **Eccentricity** - distance between ellipse foci ÷ major-axis length; 0 for a circle, increasing with elongation.
3. **Solidity** - Area ÷ Convex-hull area; values near 1 indicate a nearly convex outline, lower values indicate pronounced indentations.
4. **Extent** - Area ÷ Bounding-box area; measures how fully the shape fills its minimal enclosing rectangle.
5. **Minor-axis length** - width of the best-fitting ellipse, normalised to the same scale as the major axis (range 0–1).

Visual representatives can be observed in the supplementary data (Sup. Fig. 1).

### Data analysis

All code and models were run in Python, graphs were generated with the Matplotlib package and statistics were generated using the SciPy package. Model performance was evaluated using the F1-score, which is the harmonic mean of precision and recall and provides a balanced measure of classification performance, particularly useful for imbalanced datasets and is defined as:

$$F1=2 x \frac{Precision x Recall}{Precision + Recall}$$

## Results

### Model F1-score analysis

**Fig. 1:**
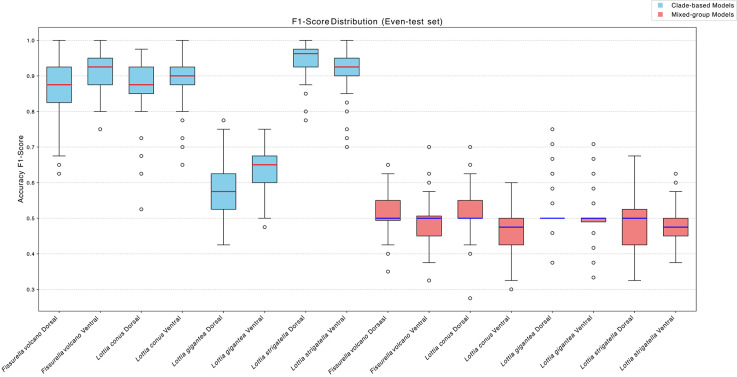
Box plots for model combination F1-scores across 100 runs for the Even-test datasets. Blue boxes are the clade-based models per species and orientation and the red boxes are the mixed-group controls. For each species and orientation, the F1-scores are significantly greater for the clade-based models compared to the mixed-group controls.

The Even-test results for clade-based models are significantly different from the mixed-group controls (Mann–Whitney U, P < 0.001; Fig. 1). Among the clade-based models, the highest median F1-scores were obtained for *L. strigatella* (dorsal: 0.963; maximum 1.000) and *F. volcano* (ventral: 0.925; maximum 1.000). The lowest performance was recorded for *L. gigantea* (dorsal: median 0.575; maximum 0.775). Mixed-group controls consistently underperformed, all of which have median values of ~ 0.500; the largest drop occurred for *F. volcano* (ventral: median 0.500; maximum 0.700).

The Full-test evaluation showed the same pattern (Sup. Fig. 2). *L. strigatella* remained the top performer (dorsal median 0.964; ventral median 0.945), whereas *L. gigantea* (dorsal median 0.575; maximum 0.688) again ranked lowest. For example, the mixed-group control for *L. conus* (ventral) achieved only 0.500 (median) and 0.576 (maximum), compared with 0.904 and 0.938 for its clade-based counterpart, confirming reliance on clade-specific features. Comparing the Even-test and Full-test scores yielded no significant difference (Kruskal–Wallis test P >0.05), indicating that overall model performance is insensitive to test-set size or class balance. All performance-based subsequent analyses therefore report the Even-test metrics and a boot-strap analysis using these 100 iterations can be seen in the supplementary data (Sup. Table 2)

### *F. volcano* morphological variation

**Fig. 2:**
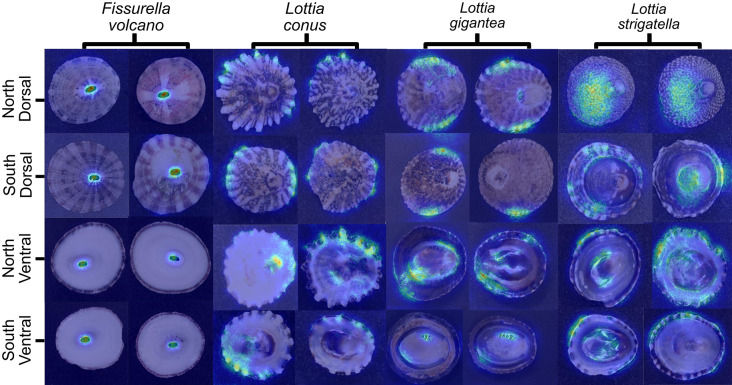
Examples of saliency maps showing all species, groups and orientations. The highlighted parts of the shells are where the model focusses attention for distinguishing between clades. *Lottia gigantea* is larger than all other species, with an average size of 45mm in length for sampled individuals. The average sizes of the sampled individuals for the other species are *L. conus* (9.7mm), *L. strigatella* (9.9 mm) and *F. volcano* (18.3 mm)

**Fig. 3:**
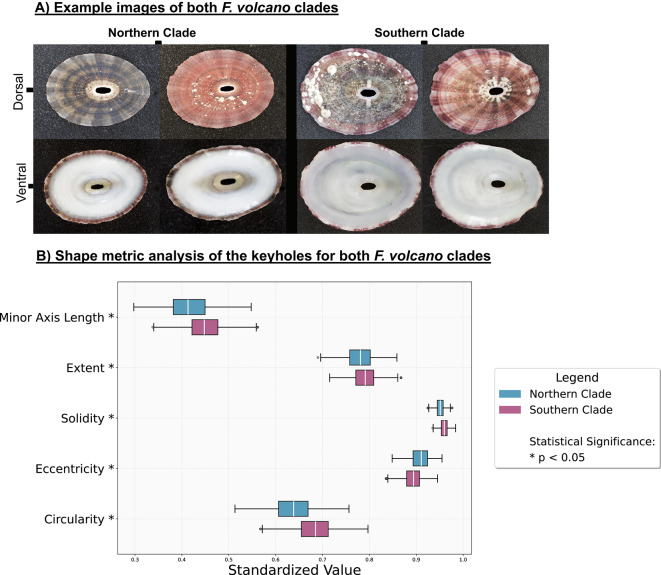
Example images of whole shell specimens from each clade and both perspectives, with the keyholes clearly visible on the apex of each shell (**A**). Shape metric analysis of *F. volcano* keyholes (**B**). The saliency maps consistently highlighted the keyholes when distinguishing between clades (see Fig 2).

Saliency mapping for both dorsal and ventral orientations consistently highlighted the keyhole (Fig. 2). We therefore selected this structure for detailed, mask-based shape analysis. All five metrics—circularity, eccentricity, solidity, extent and minor-axis length—differed significantly between the Northern and Southern clades (Mann–Whitney U, P < 0.001; Fig. 3b). Northern keyholes were less circular, more elongated, more indented, filled a smaller proportion of their bounding box and had a shorter minor axis, whereas Southern keyholes showed the opposite pattern.

**Fig. 4:**
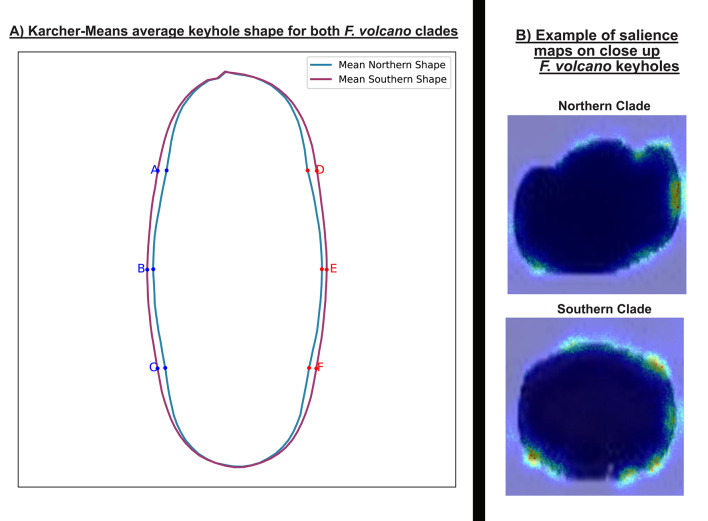
Karcher-mean depiction of the mean keyhole shape of the two *F. volcano* clades (**A**) and saliency map examples conducted on just the keyholes (**B**). The keyholes of specimens from the northern clade are more indented and less circular compared to the keyholes of specimens from southern clades*.*

To further quantify these differences in keyhole shape, we compared the average outlines of the keyholes in each clade using the Karcher-mean. A Karcher-mean represents the average shape derived from all specimen examinations, providing a single outline that best captures the overall form while accounting for variation among individual shapes. A Karcher-mean shape comparison reinforced these differences (Fig. 4a). Six corresponding outline points were defined along the keyhole margin at the 25th, 50th, and 75th percentiles of maximum height on both left and right sides of the keyhole, ensuring consistent spatial correspondence across specimens for subsequent alignment and shape comparison. These showed that the Northern keyhole is 12.0% narrower along the minor axis, and the mean upper- and lower-quartile distances are 42.5% greater than the central distance, emphasising its more irregular outline. Location-specific Karcher-means display the same clade-level contrast (Sup. Fig. 3).

When the keyhole region (plus a small bounding margin) was cropped from every image and re-submitted to the classifier, the median F1-score fell to ~0.70. When looking at just the keyholes, salience maps were centred on the keyhole perimeter, particularly where inter-clade shape differences occur (Fig. 4b), indicating that the keyhole is the principal, but not sole, feature underpinning discrimination.

### *L. conus* morphological variation

**Fig. 5:**
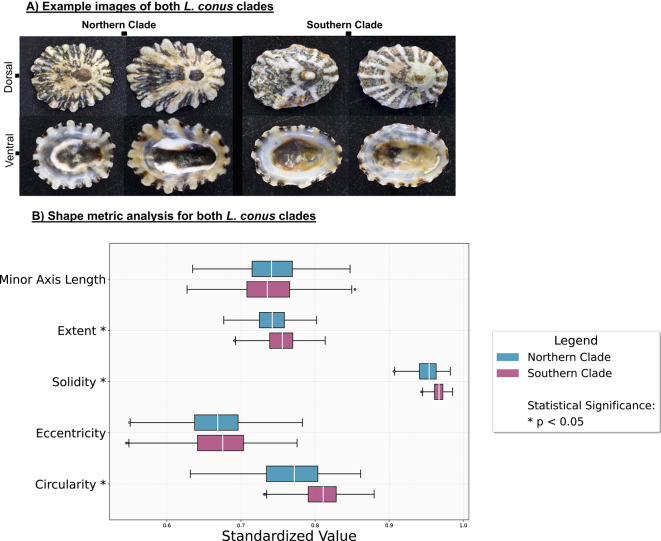
Example images of shells from both *L. conus* clades and perspectives (**A**) and shape metric analysis of *L. conus* shells (**B**).

Saliency maps for *L. conus* converged on the distal ridge tips in both dorsal and ventral orientations (Fig. 2). Guided by this pattern we compared whole-shell outlines between clades. Masks were generated for every shell, rescaled to a common major-axis length, and the standard shape metrics extracted. Circularity, solidity and extent differed significantly between the Northern and Southern clades (Mann–Whitney U, P < 0.001), whereas eccentricity and minor-axis length showed no significant variation (Fig. 5b). Thus, Northern shells are on average more irregular, have deeper indentations and occupy a smaller proportion of their bounding box than Southern shells, while overall elongation remains comparable.

### *L. gigantea* and *L. strigatella* morphological variation

**Fig. 6:**
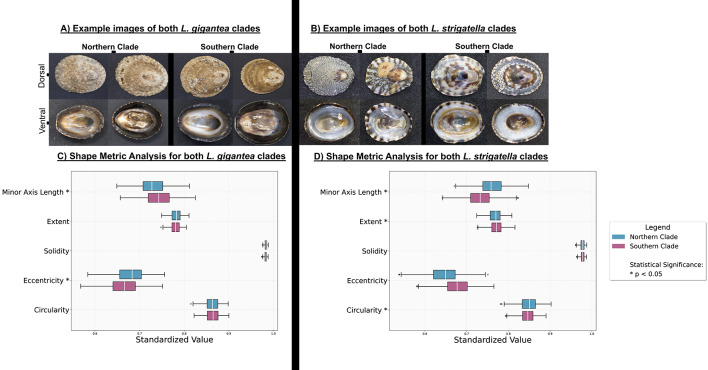
Example images of shells from both *L. gigantea* clades and perspectives (**A**) and *L. strigatella* (**B**). Shape metric analysis of *L. gigantea* (**C**) and *L. strigatella* (**D**) shells.

For *L. gigantea* and *L. strigatella* the saliency maps were diffuse, with attention often distributed along the shell perimeter rather than on a single, discrete structure (Fig. 2). Consequently, we applied the same whole-shell, mask-based shape analysis to these species to quantify any outline differences between clades (Fig. 6.

For *L. gigantea,* the Northern clade shells were less elongated (lower eccentricity) and displayed a larger normalised minor-axis length than those of the Southern clade, whereas circularity, solidity and extent did not differ significantly. For *L. strigatella*, the Northern clade shells were more regular in outline (higher circularity), had a larger minor-axis length and a lower extent (less compact) than Southern shells, while solidity was nonsignificant between clades.

## Discussion

### CV as a powerful tool for biodiversity research

Understanding and preserving biodiversity requires advanced methods to detect and analyse cryptic morphological diversity among genetically distinct clades^39^. In this study, we explored the potential of explainable artificial intelligence, specifically CV, to reveal cryptic morphological divergence among genetically distinct clades of limpets. Our findings demonstrate that CV methods can not only accurately classify individuals from four limpet species into their respective genetic clades (Fig. 1) but also identify and quantify morphological features of clades that were yet to be recognised or reported by human observers. The successful application of CV in this context underscores its value as a powerful tool for biodiversity research, providing new insights into the eco-evolutionary processes shaping morphological traits within cryptically diverse species.

Although demonstrated here with limpet species, the workflow is transferable because it operates on two-dimensional images in which the relevant diagnostic information lies within a single plane. Numerous taxonomic groups satisfy this criterion, including, but not limited to, insect wings^40^ and other pinned or slide-mounted materials^41^, herbarium sheets^42^, and thin-section microfossils^43^. Major natural history repositories, including the Natural History Museum, London, are now digitising such objects at scale, thereby supplying abundant datasets for further investigation^12,44,45^. Researchers wishing to adopt the pipeline therefore need only modest adjustments. For example, substitute an appropriate training image set, fine-tune the hyper-parameters, and rerun the training–validation cycle on a pre-weighted convolutional network. When coupled with heatmap-guided feature visualisation and mask-based shape analysis, which are both easily automated with contemporary detection and segmentation models^37,38^, this approach enables the objective localisation and quantification of defining characters in any planar specimen. Although image augmentation introduced some positional variation, all images were captured under controlled and standardised conditions, with the camera positioned directly above each specimen at a fixed distance and lighting setup. Such consistency is critical for reliable feature detection, as differences in angle, illumination, or scale could alter the appearance of diagnostic features and reduce model reproducibility. Assessing how well the method performs under less standardised imaging conditions would therefore be a valuable future test.

### Detecting clade-specific signals

Comparisons between the clade-based models and the mixed-group controls show that the pipeline is responding to genuine, clade-specific signals. Mixed-group controls have significantly lower F1-scores (F1 = ~0.5). This means their performance is similar to random guessing for problems with two classes^25^. This confirms that F1-score performance in the original models is driven by clade-specific characters rather than generic image features^46,47^. These characters, though subtle, are evidently consistent enough to support reliable automated discrimination. Most original configurations achieved very high F1-scores, and several runs reached perfection (F1-score = 1.0) for both dorsal and ventral views of *L. strigatella* and *F. volcano,* while the *L. strigatella* dorsal perspective reached an average F1-score of 0.96 across its 100 iterations. The F1-score performance for *L. gigantea* was appreciably lower (Fig. 1). The smaller difference in F1-scores between clade-based and mixed-group models potentially reflects a relatively recent population divergence in this species. Genome-wide SNP data indicate a population split, but *COI* data show no corresponding division, suggesting that perhaps this separation is evolutionarily young. Such discordance between nuclear and mitochondrial markers often occurs during early stages of population isolation, when genomic differentiation emerges before fixed morphological or mitochondrial differences accumulate^48,49^. The absence of pronounced morphological divergence in *L. gigantea* therefore implies that while this species may have some geographically structured genomic divergence, where selection or restricted gene flow has begun to structure genomic variation, phenotypic differentiation has yet to accumulate. This contrasts with the other species, where older, mitochondrial-level separations coincide with measurable morphological divergence, producing higher F1-scores for clade-based models. Note however, our clades for *L. gigantea* were only based on the Californian phylogeographic break which is less clear geographically (in the Los Angeles region) and with lower support than the more distinct break in the central Baja California Peninsula^14^. If more specimens were available for this southernmost clade, the models might have performed better. In addition, the average length of the *L. gigantea* shells analysed here was 44.6 mm, significantly larger than those of the other species: *L. conus* (9.7mm), *L. strigatella* (9.9 mm) and *F. volcano* (18.3 mm). *Lottia gigantea* individuals frequently exhibit pronounced shell erosion^50,51^, which may remove some details.

The absence of any significant difference between the Even-test and Full-test evaluations confirms that the morphological signal is independent of sample size or class imbalance. F1-scores nevertheless showed variable dispersion across their respective iterations: the broadest range (0.450) arose in the *L. conus* dorsal models, whereas the narrowest (0.225) was recorded for *L. strigatella* dorsal models, indicating a more stable response in the latter. Variation of this kind is often linked to dataset quality^52,53^. Consistent imaging protocols and balanced classes are critical to reliable performance^54–56^, yet limpet shells are inherently variable and may be eroded, damaged or obscured by surface deposits^13^. Although severely damaged specimens were removed, residual heterogeneity remained, so some random training–validation splits inevitably contained fewer informative features. Implementing 100 independent resampling iterations was therefore essential for exposing and averaging over this variance, and similar protocols are recommended when benchmarking image classification pipelines.

### Relevant characters for cryptic morphological divergence

Saliency mapping highlighted the image regions that most influenced the classifier and thus pointed to characters of possible ecological or evolutionary relevance. In *F. volcano*, the maps converged on the keyhole; shape metrics confirmed significant clade differences in circularity, eccentricity, solidity, extent and minor-axis length, with Northern keyholes narrower and more indented than Southern ones. When images were cropped to the keyhole alone, saliency shifted to the aperture perimeter and the F1-score fell to ~0.70, indicating that the keyhole is the principal, but not exclusive, discriminant. Importantly, these clade-specific shapes can be observed at each location (i.e., specimens from each Northern location had keyholes that are narrower and indented and specimens from each Southern location are more oval shaped). This suggests that these morphological differences are clade-specific and therefore likely related to the genetic differences between clades^15^, but further research is required.

For *L. conus*, the maps consistently highlighted the ridge tips. Corresponding shape analyses showed that Northern shells were more irregular, had lower solidity and occupied a smaller proportion of their bounding box, signifying greater concavity relative to Southern shells. In *L. gigantea* and *L. strigatella* the saliency maps were less convergent, with attention dispersed across the shell surface and occasionally concentrated along the perimeter. Even so, quantitative metrics detected clear clade-level shell-based divergence. *Lottia gigantea* clades differed significantly in eccentricity and minor-axis length, whereas *L. strigatella* clades diverged in circularity, extent and minor-axis length. The diffuse saliency pattern implies that the discriminating information is spread across the shell or resides in attributes not captured by outline geometry, such as colour bands or surface patterning. This interpretation is supported by the exceptionally strong and stable performance of the *L. strigatella* models, whose high mean F1-score and narrow dispersion suggest additional, non-geometric cues underpin effective classification in that species. While many of the highlighted regions correspond to biologically interpretable shell features, not all saliency responses necessarily reflect genuine morphological signal. Saliency maps identify regions that most strongly influence model decisions rather than features of confirmed biological relevance, and activation can occasionally arise from background texture, residual reflections, or minor lighting differences. This limitation is inherent to most image-based explainability methods and should be considered when interpreting fine-scale patterns of activation. Furthermore, AI and CV models operate purely as mathematical systems that detect and process numerical patterns; they do not possess an intrinsic understanding of the biological meaning of these patterns. Nevertheless, the overall consistency of highlighted regions across models and orientations suggests that the major areas of importance are clade specific rather than stochastic or artefactual.

The adaptive drivers of the clade-level differences documented here remain unresolved. Furthermore, we do not currently know whether morphological differences between clades are a result of genetic differences or due to phenotypic plasticity (i.e., caused by differences in environmental or ecological conditions between clades). Morphological traits generally evolve under selective pressures arising from environmental conditions, resource acquisition or predation^57–59^. In Fissurellidae, the keyhole serves as an exhalant opening for waste or respiration^60^. A latitudinal survey across its congener (*F. radiosa*) shows that the keyhole narrows towards the cooler portion of its geographic range (albeit with limited spatial sampling) ^65^. While clade differences in keyhole shape detected in *F. volcano* generally matches this trend, the precise advantage of keyhole shape remains to be studied empirically. Nor is it known whether keyhole shape is a result of the genetic differences between clades, phenotypic plasticity (i.e., caused by water temperature or other environmental differences between locations/regions) or a combination of both. For *L. conus*, comparable data are limited. Some studies suggest that limpet shell morphology is shaped by the need to maintain attachment to the substrate, influenced by factors such as wave exposure and the physical characteristics of the surface^61,62^. However, these studies note that limpet shells can become more or less conical under different environmental regimes but do not explain why the ridge tips themselves become more elongated and projecting, rather than remaining broadly rounded.

Future research could explore many extensions to the current framework. One practical direction would be to test the classifier on images captured under varying camera angles and lighting conditions to assess how robust the workflow remains under less standardised imaging. Beyond this, future developments in three-dimensional imaging could overcome such limitations by recording complete shell geometry, allowing morphological differences to be examined independently of viewing angle. For instance, three-dimensional imaging that records shell height and curvature would permit finer quantification of morphological divergence and would allow research into subjects that do not sit on a single plane. Morphology-based assessments that incorporate colour or surface-pattern information^63^, rather than geometry alone, could expose additional clade-specific characters. More precisely linking morphological differences identified by AI methods to genetic differentiation through targeted genomic sampling could illuminate how genotype-phenotype interactions contribute to observed morphological variation^64^, potentially identifying specific genomic regions that underlie morphological divergence between clades. Together, these advances should deepen our understanding of the trait variation driving evolutionary differentiation and enhance conservation assessments of cryptic biodiversity.

## Conclusion

This study highlights how computer vision, combined with user-friendly AI tools like saliency mapping, can uncover hidden patterns in shapes and forms between cryptic groups. This approach not only enhances our understanding of diversity in nature but also makes complex analysis easier and more precise. The pipeline reliably classified individuals into their genetically defined clades and pinpointed clade-specific shell characters, such as the keyhole in *F. volcano* and the ridge-tip geometry in *L. conus*, demonstrating that it is driven by clade-specific features rather than irrelevant image cues. Although this analysis does not pinpoint the exact reasons behind these differences, it provides a useful framework for exploring subtle variations in any group of organisms, especially when their key characteristics are primarily presented in two dimensions. As natural history collections continue to release large image datasets, the scope for applying explainable AI workflows across diverse organismal groups will grow correspondingly.

## Supplementary Information

Supplementary Information.

## Data Availability

Data for this project is available at:https://github.com/JackDanHollister/chapter_3-_genes_shells_and_AI_data.
