# Supplementary material for: Genes, shells, and AI: using computer vision to detect cryptic morphological divergence between genetically distinct populations of limpets
Source: Sci Rep. 2025 Dec 12;16:1051. doi: 10.1038/s41598-025-30613-1 (PMC12783718; doi:10.1038/s41598-025-30613-1)
Supplement: Supplementary file 1 — Supplementary Information. [file 41598_2025_30613_MOESM1_ESM.docx]

**Supplementary data**

Supplementary figure 1. Visual representations of the shape metrics

Supplementary figure 2. Box plots for model combination F1-scores across 100 runs for the full-test datasets. Blue boxes are the clade-based models per species and orientation and the red boxes are the mixed-group controls. For each species and orientation, the F1-scores are significantly greater for the clade-based models compared to the mixed-group controls.


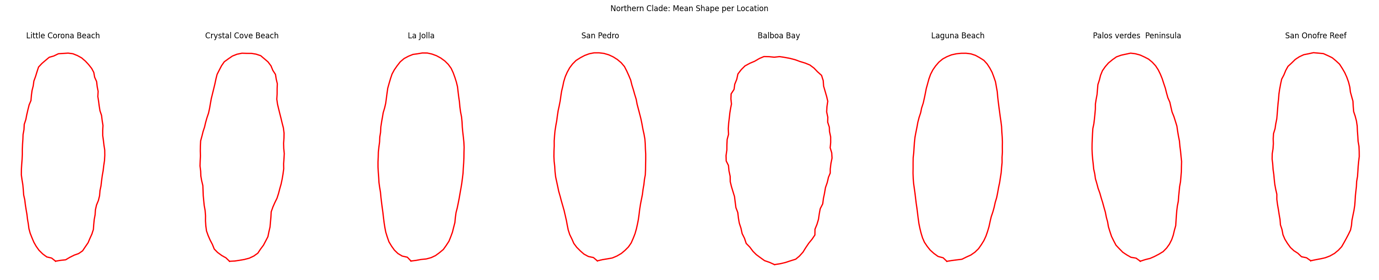


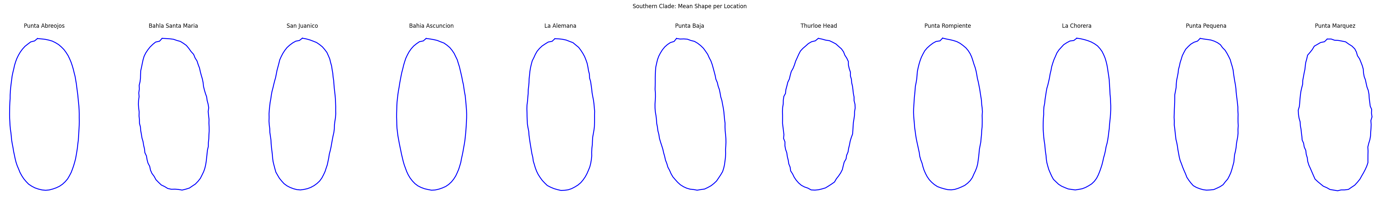


Supplementary figure 3. Karcher-means shape for all locations of *Fissurella volcano* specimens

Supplementary table 1. Number of limpets sampled per location and clade allocations per species (FV = *Fissurella volcano*; LC = *Lottia conus*; LG = *Lottia gigantea*; LS = *Lottia strigatella*). All specimens sampled in the field were collected in 2023. LACM is the Los Angeles County Museum of Natural History.

| Species | Location | Sampled | Number | Clade | Latitude | Longitude |
| --- | --- | --- | --- | --- | --- | --- |
| FV | Balboa Bay | LACM | 2 | North | 33.58 | -117.86 |
| FV | Crystal Cove Beach | LACM | 11 | North | 33.57 | -117.84 |
| FV | La Jolla | LACM | 33 | North | 32.83 | -117.28 |
| FV | Laguna Beach | LACM | 39 | North | 33.54 | -117.79 |
| FV | Little Corona Beach | LACM | 6 | North | 33.59 | -117.87 |
| FV | Palos verdes Peninsula | LACM | 27 | North | 33.74 | -118.42 |
| FV | San Onofre Reef | LACM | 11 | North | 33.37 | -117.56 |
| FV | San Pedro | LACM | 52 | North | 33.70 | -118.29 |
| FV | Bahia Ascuncion | Field | 95 | South | 27.12 | -114.26 |
| FV | La Chorera | Field | 32 | South | 30.47 | -116.06 |
| FV | Bahla Santa Maria | LACM | 8 | South | 24.76 | -112.27 |
| FV | La Alemana | Field | 17 | South | 29.95 | -115.75 |
| FV | Punta Abreojos | Field | 109 | South | 26.70 | -113.55 |
| FV | Punta Baja | Field | 16 | South | 29.95 | -115.81 |
| FV | Punta Marquez | LACM | 3 | South | 23.96 | -110.87 |
| FV | Punta Pequena | LACM | 17 | South | 26.23 | -112.50 |
| FV | Punta Rompiente | LACM | 38 | South | 27.68 | -114.93 |
| FV | San Juanico | Field | 32 | South | 26.23 | -112.50 |
| FV | Thurloe Head | LACM | 4 | South | 27.62 | -114.85 |
| LC | Encinitas | LACM | 4 | North | 33.04 | -117.30 |
| LC | San Pedro | LACM | 39 | North | 33.70 | -118.29 |
| LC | La Jolla | LACM | 9 | North | 32.83 | -117.28 |
| LC | Lunada Bay | LACM | 7 | North | 33.77 | -118.43 |
| LC | Venice | LACM | 25 | North | 33.97 | -118.45 |
| LC | Santa Monica | LACM | 32 | North | 34.00 | -118.49 |
| LC | Campo Kennedy | Field | 85 | North | 31.70 | -116.68 |
| LC | La Chorera | Field | 41 | North | 30.47 | -116.06 |
| LC | UABC | Field | 59 | North | 31.86 | -116.67 |
| LC | Punta Baja | Field | 44 | North | 29.95 | -115.81 |
| LC | Bahia Ascuncion | Field | 100 | South | 27.12 | -114.26 |
| LC | Punta Abreojos | Field | 152 | South | 26.70 | -113.55 |
| LC | San Juanico | Field | 344 | South | 26.23 | -112.50 |
| LC | Millers Landing | LACM | 2 | South | 28.47 | -114.05 |
| LC | Punta Abreojos | LACM | 3 | South | 26.70 | -113.55 |
| LC | Punta Pequena | LACM | 3 | South | 26.23 | -112.50 |
| LC | Punta Rompiente | LACM | 25 | South | 27.72 | -115.02 |
| LG | Topanga | LACM | 6 | North | 34.03 | -118.61 |
| LG | Hazard Canyon | LACM | 3 | North | 35.29 | -120.88 |
| LG | Pacific Grove | LACM | 16 | North | 36.62 | -121.92 |
| LG | Crescent City | LACM | 10 | North | 41.75 | -124.20 |
| LG | Granite Creek | LACM | 6 | North | 36.43 | -121.91 |
| LG | Waddell Beach | LACM | 2 | North | 37.12 | -122.28 |
| LG | Santa Monica | LACM | 13 | North | 34.00 | -118.49 |
| LG | Franklin Point | LACM | 1 | North | 37.65 | -86.87 |
| LG | Venice | LACM | 35 | North | 33.97 | -118.45 |
| LG | Santa Barbara | LACM | 8 | North | 34.42 | -119.70 |
| LG | Morro Bay | LACM | 27 | North | 35.33 | -120.85 |
| LG | Morro Rock | LACM | 3 | North | 35.33 | -120.87 |
| LG | Pt Dume | LACM | 27 | North | 34.00 | -118.81 |
| LG | Pt Reyes | LACM | 1 | North | 38.00 | -122.99 |
| LG | Venice Breakwater | LACM | 3 | North | 33.97 | -118.45 |
| LG | San Mateo | LACM | 1 | North | 37.65 | -122.33 |
| LG | San Pedro | LACM | 53 | South | 33.70 | -118.29 |
| LG | Palos Verdes Peninsula | LACM | 46 | South | 33.74 | -118.42 |
| LG | Laguna Beach | LACM | 13 | South | 33.54 | -117.79 |
| LG | Palos Verdes | LACM | 12 | South | 33.74 | -118.42 |
| LG | Pt Vncente | LACM | 9 | South | 33.74 | -118.41 |
| LG | Bahia Rosarita | LACM | 9 | South | 32.35 | -117.04 |
| LG | Hermosa Beach | LACM | 9 | South | 33.86 | -118.39 |
| LG | Arbolitos | LACM | 7 | South | 31.70 | **- 118.39** |
| LG | Coronado Island | LACM | 6 | South | 32.42 | -117.29 |
| LG | Newport Bay | LACM | 6 | South | 33.60 | - 117.88 |
| LG | Descanso | LACM | 5 | South | 32.20 | - 116.61 |
| LG | Abalone Point | LACM | 4 | South | 33.55 | - 117.81 |
| LG | Santa Cruz | LACM | 3 | South | 36.95 | - 122.02 |
| LG | San Pedro Breakwater | LACM | 3 | South | 33.72 | -118.30 |
| LG | Victoria Cove | LACM | 3 | South | 33.52 | -119.41 |
| LG | North of Ensenada | LACM | 2 | South | 31.87 | -116.60 |
| LS | Bahia Ascuncion | Field | 53 | North | 27.12 | -114.26 |
| LS | La Alemana | Field | 30 | North | 29.95 | -115.75 |
| LS | Punta Abreojos | Field | 5 | North | 26.70 | -113.55 |
| LS | Campo Kennedy | Field | 24 | North | 31.70 | -116.68 |
| LS | Punta Baja | Field | 41 | North | 29.95 | -115.81 |
| LS | San Juanico | Field | 47 | North | 26.23 | -112.50 |
| LS | UABC | Field | 15 | North | 31.86 | -116.67 |
| LS | Pozo De Cota | Field | 111 | South | 23.02 | -110.10 |
| LS | Punta Marquez | Field | 463 | South | 23.96 | -110.87 |

Supplementary table 2. Bootstrap analysis of the 100 iterations for each of the 16 examinations.

| **examination** | **n_iterations** | **accuracy_median** | **accuracy_2.5%** | **accuracy_97.5%** | **macro_f1_median** | **macro_f1_2.5%** | **macro_f1_97.5%** |
| --- | --- | --- | --- | --- | --- | --- | --- |
| F.volcano_dorsal | 100 | 0.875 | 0.65 | 0.95 | 0.874 | 0.601 | 0.95 |
| F.volcano_ventral | 100 | 0.925 | 0.825 | 1 | 0.925 | 0.819 | 1 |
| L.conus_dorsal | 100 | 0.875 | 0.699 | 0.975 | 0.875 | 0.674 | 0.975 |
| L.conus_ventral | 100 | 0.9 | 0.749 | 0.975 | 0.899 | 0.738 | 0.975 |
| L.gigantea_dorsal | 100 | 0.575 | 0.45 | 0.7 | 0.561 | 0.366 | 0.7 |
| L.gigantea_ventral | 100 | 0.65 | 0.512 | 0.738 | 0.631 | 0.469 | 0.733 |
| L.strigatella_dorsal | 100 | 0.962 | 0.824 | 1 | 0.962 | 0.818 | 1 |
| L.strigatella_ventral | 100 | 0.925 | 0.774 | 1 | 0.925 | 0.763 | 1 |
| F.volcano_dorsal_mixed | 100 | 0.5 | 0.4 | 0.625 | 0.479 | 0.316 | 0.621 |
| F.volcano_ventral_mixed | 100 | 0.5 | 0.375 | 0.588 | 0.431 | 0.315 | 0.562 |
| L.conus_dorsal_mixed | 100 | 0.5 | 0.425 | 0.7 | 0.373 | 0.31 | 0.69 |
| L.conus_ventral_mixed | 100 | 0.475 | 0.337 | 0.538 | 0.333 | 0.298 | 0.52 |
| L.gigantea_dorsal_mixed | 100 | 0.5 | 0.415 | 0.667 | 0.333 | 0.316 | 0.644 |
| L.gigantea_ventral_mixed | 100 | 0.5 | 0.375 | 0.625 | 0.333 | 0.304 | 0.608 |
| L.strigatella_dorsal_mixed | 100 | 0.5 | 0.337 | 0.625 | 0.406 | 0.304 | 0.584 |
| L.strigatella_ventral_mixed | 100 | 0.475 | 0.4 | 0.6 | 0.43 | 0.316 | 0.591 |
